# Promotion of health-enhancing physical activity in the sport sector: a study among representatives of 536 sports organisations from 36 European countries

**DOI:** 10.1186/s12889-023-15589-9

**Published:** 2023-04-24

**Authors:** Tena Matolić, Danijel Jurakić, Hrvoje Podnar, Ivan Radman, Željko Pedišić

**Affiliations:** 1 Faculty of Kinesiology, University of Zagreb, Horvaćanski zavoj 15, Zagreb, 10110 Croatia; 2 Institute for Health and Sport, Victoria University, Footscray Park Campus, Ballarat Road, Footscray, VIC 3011 Australia

**Keywords:** Europe, Health-enhancing sports, Physical activity, Sports association, Sports club, Sports Club for Health Guidelines

## Abstract

**Background:**

It is a common belief that most sports clubs and organisations are primarily focused on elite sports while placing less emphasis on the promotion of health-enhancing physical activity (HEPA). However, there is a lack of evidence on this topic in the scientific literature. Therefore, the aim of this study was to determine the level and correlates of the commitment of sports organisations in Europe to HEPA promotion.

**Methods:**

Representatives of 536 sports organisations from 36 European countries responded to our survey. A multiple regression analysis was conducted with the commitment of sports organisation to HEPA promotion (0 [“not at all”] – 10 [“most highly”]) as the outcome variable and organisation type (“national sport association” reference group [ref], “European sports federation”, “national umbrella sports organisation”, “national Olympic committee”, “national sport-for-all organisation”), headquarters in a European Union member state (“no” [ref], “yes”), region of Europe (“Western” [ref], “Central and Eastern”, “Northern”, “Southern”), commitment to elite sports (“low” [ref], “medium”, “high”), and awareness of Sports Club for Health (SCforH) guidelines (“no” [ref], “yes”) as explanatory variables.

**Results:**

Approximately 75.2% (95% confidence interval [CI]: 71.5, 78.8) of sports organisations were highly committed to elite sports. Only 28.2% (95% CI: 24.4, 32.0) of sports organisations reported a high commitment to HEPA promotion. A higher commitment to HEPA promotion was associated with the national Olympic committees (*β* = 1.48 [95% CI: 0.41, 2.55], *p* = 0.007), national sport-for-all organisations (*β* = 1.68 [95% CI: 0.74, 2.62], *p* < 0.001), location in Central and Eastern Europe (*β* = 0.56 [95% CI: 0.01, 1.12], *p* = 0.047), and awareness of SCforH guidelines (*β* = 0.86 [95% CI: 0.35, 1.37], *p* < 0.001).

**Conclusion:**

From our findings, it seems that most sports organisations are primarily focused on elite sports. Coordinated actions at the European Union and national levels are needed to improve the promotion of HEPA through sports organisations. In this endeavour, it may be useful to consider national Olympic committees, national sport-for-all organisations, and relevant sports organisations in Central and Eastern Europe as role models and to raise the awareness of SCforH guidelines.

## Background

Physical activity has a wide range of benefits for health and well-being [1]. It reduces the risk of various chronic diseases, such as coronary heart disease, type 2 diabetes, metabolic syndrome, obesity, and several types of cancer [1]. Even just one hour of moderate-intensity physical activity per week is associated with a 33% lower risk of mortality [2]. Despite these benefits and global efforts to promote physical activity, the global prevalence of not meeting the recommended levels of physical activity is still very high; approximately 27.5% among adults [3] and 81% among adolescents [4]. Physical activity promotion is, therefore, one of the key public health priorities globally.

Different settings provide opportunities to engage in physical activity, with sports clubs being among the most represented ones [5]. While common reasons for participation in sports are enjoyment, social interactions, and weight management [6], sports club members may also be elite athletes focused on training at a high load and achieving top-level results in competition [7]. In this paper, we generally refer to sports participation for recreational purposes.

Epidemiological research has shown a range of health benefits associated specifically with recreational sports participation, including improved aerobic and metabolic fitness, improved cardiovascular function at rest, reduced adiposity, reduced risk of all-cause mortality, and improved psychological health and social well-being [8–11]. The individuals who play sports in a sports club are more likely to regularly engage in physical activity than others [12–14], and the participation in sports activities, therefore, significantly contributes to achieving recommended levels of physical activity [13, 15, 16]. Other benefits of sports for the society include better integration of minorities [17] and people with disabilities [18], as well as improved socialisation of older adults, children, and adolescents [8].

The implementation of sports programmes in the community is considered as one of the “best investments” for population health [19]. A study conducted in England suggested that encouraging participation in activities of higher intensity among females, preventing reduction in exercise intensity associated with ageing among males, and providing adequate facilities are key policy challenges for HEPA promotion through sports [20].The sports clubs may play an important role in addressing these and other challenges in health promotion, because of their high population reach [21, 22] and a range of health benefits associated with sports club participation [14, 23]. Therefore, sports clubs are deemed as a suitable setting for HEPA promotion [5, 24].

In some countries, such as the United Kingdom, sport and physical activity policies seem to have a twofold focus on top-level performance in competitions and ‘active citizens’ [25]. Activities that generate more economic benefits are likely to receive more funding, and elite sport is often perceived as more “valuable” in this regard [25, 26]. Such perception may facilitate the development of professional sports clubs [27], while limiting opportunities for mass sport participation. Complementarity between elite sport development and the promotion of ‘sport for all’ is often discussed, especially at the political level [28] but it should not necessarily be assumed. Even in countries with national policies that promote such complementarity, sports clubs and organisations at the grass-root level may encounter a range of difficulties when trying to achieve and maintain a good balance between elite sports development and HEPA promotion, such as lack of funding, inadequate facilities and equipment, shortage of staff and volunteers, and insufficient “how-to” knowledge [5, 14, 18, 28–30].

To help overcome these difficulties, the largest European Union (EU) initiative for the promotion of HEPA through sports clubs—*Sports Club for Health* (SCforH)—has been in place since 2008. The principles of the SCforH approach and recommended steps for its implementation in sports clubs have been described in the SCforH guidelines [5], textbook [31], and online course. In 2013, the Council of the EU recognised the importance of implementing the SCforH guidelines in sports clubs and listed it as one of 23 indicators for evaluation of health-enhancing physical activity (HEPA) promotion in the EU countries. In the White Paper on Sport, the European Commission supported the promotion of sports to achieve a healthy society and emphasised the importance of HEPA promotion as an integral part of sports organisations [32]. Despite the recognition of sports clubs as an important setting for HEPA promotion at the highest political level in the EU [33–35], a recent study found that only 12% of EU citizens are involved in sports and recreational activities within sports clubs [36].

It is widely considered that most sports clubs and organisations are primarily focused on elite sports and achieving top results in competitions, while placing less emphasis on sport-for-all and HEPA in general [12, 17, 28–30]. However, no recent quantitative evidence is available to corroborate this widespread assumption, and the actual commitment of sports clubs and organisations to HEPA remains to be elucidated. Such evidence is important from a public health perspective, as it would inform future HEPA promotion policies and initiatives in the sports sector. Therefore, the aim of this paper was to explore the level and correlates of commitment of sports organisations in Europe to promoting HEPA.

## Methods

### Study design and participants

In 2016/17, we conducted a questionnaire-based, cross-sectional study among representatives of sports organisations from 36 European countries, including 28 EU member states at the time, 4 candidate countries (Albania, North Macedonia, Serbia, and Turkey), Iceland, Monaco, Norway, and Switzerland. Our study sample did not include regional- and local-level organisations. Out of 1717 invited representatives of sports organisations, 536 agreed to participate in the study and responded to the survey. All participants gave informed consent before responding to the survey. The sample included representatives of: European umbrella sports organisations, national Olympic committees, national sport associations, national sport-for-all organisations, and national umbrella sports organisations. Sample characteristics are presented in Table 1. The study protocol was approved by the Scientific and Ethics Committee of the University of Zagreb, Faculty of Kinesiology (ref: 102/2016).

### Measures

We collected the following data in relation to the participating sports organisations: the type of organisation, the country in which their headquarters are located, the awareness of SCforH guidelines among their representatives, and their level of commitment to promoting different types of physical activity. The awareness of SCforH guidelines was assessed with the question *“Prior to this survey, as a representative of your sports organisation, were you aware of the ‘Sports Club for Health Guidelines’?”*. The level of commitment to promoting different types of physical activity data was assessed with the questions: “*Please estimate how much is your sports organisation committed to the promotion of:”* (a) *“Elite sports”*, (b) *“Health-enhancing sports, recreational sports or ‘sport for all’”*, (c) *“Health-enhancing exercise (for example, Nordic walking, aerobics, gym workout)”*, and (d) *“Health-enhancing lifestyle physical activities (for example, gardening, walking or cycling for transport, stair climbing)”*, with the response scale from 0 (“*Not at all*”) to 10 (“*Most highly*”). The questions were developed through discussion between three authors (ZP, HP, and IR), and their *a priori* validity was confirmed by 11 experts in physical activity research and promotion, members of the SCforH Consortium. Based on the responses to these four questions, we created two summary variables: commitment to the promotion of elite sports (question “a”) and commitment to HEPA promotion (calculated as the arithmetic mean of responses to the questions b, c, and d), with satisfactory inter-rater reliability (intraclass correlation coefficient [ICC] = 0.72 and 0.81, respectively). We additionally determined the EU membership and region of Europe in which the organisation is located. According to EuroVoc [37], we classified the countries into four regions: Central and Eastern, Western, Southern, and Northern Europe.

### Data analysis

We calculated percentages and their 95% confidence intervals (CIs) for “low” (0–3), “medium” (4–6), and “high” (7–10) levels of commitment to HEPA promotion in the overall sample and stratified by the type of organisation, country membership in the EU, region of Europe, commitment to elite sports, and the awareness of SCforH guidelines. Fisher’s exact test was used to test the difference between levels of commitment of sports organisations to HEPA promotion across the strata. The categorisation of commitment to HEPA into “low”, “medium”, and “high” was used only for the descriptive purposes and tests of differences.

The multiple linear regression analysis was used to examine the relationships between the level of commitment to the promotion of HEPA expressed on the scale from 0 to 10 (dependent variable) and the type of organisation (reference group [ref] = national sport associations), commitment to the promotion of elite sports categorised as “low” (0–3), “medium” (4–6), and “high” (7–10) commitment (ref = “*low commitment*”), EU membership (ref = non-member), region of Europe (ref = Western), and the awareness of SCforH guidelines (ref = “*No*”). We presented unstandardized regression coefficients alongside their 95% confidence intervals (CIs) and p-values. The regression model was checked for normality of residuals using the normal probability plot, for multicollinearity using the variance inflation factors, and for heteroscedasticity using the predicted vs. residuals plot. The statistical significance was tested at *p* < 0.05.

Additionally, we conducted three multiple ordinal logistic regression (proportional odds) analyses, with the above-mentioned set of independent variables and the commitment to the promotion of: (i) health-enhancing sports activity; (ii) health-enhancing exercise; and (iii) health-enhancing lifestyle physical activities as outcome variables. The dependent variables in these analyses were expressed on the scale from 0 to 10. The ordinal logistic regression analyses were conducted because the multiple linear regression models with these three dependent variables did not meet assumptions for linear regression analysis, particularly in regard to the normality of residuals. For each ordinal regression model, we assessed proportional odds assumption and goodness of fit using the Hosmer-Lemeshow, Brant, Lipsitz, and Pulkstenis-Robinson tests. The descriptive analyses, Fisher’s exact tests, and multiple linear regression analysis were performed using RStudio (version 1.4.1103) with “stats” [38], “pastecs” [39], and “performance” [40] packages. The ordinal regression analyses were performed in RStudio (version 2022.12.0 + 353 “Elsbeth Geranium” Release) with “MASS” [41], “brant” [42], and “generalhoslem” [43] packages.

## Results

Approximately three out of four (75.2% [95% CI: 71.5, 78.8]) sports organisations reported a high commitment to elite sports. Less than one third (28.2% [95% CI: 24.4, 32.0]) of sports organisations reported a high commitment to HEPA promotion (Table 1). We found significant (unadjusted) differences in the commitment to HEPA promotion by the type of organisation (*p* < 0.001), the level of commitment to elite sports (*p* = 0.031), and the awareness of SCforH guidelines (*p* < 0.001). The highest percentage of sports organisations with a low commitment to HEPA promotion was found among national sport associations (34.8% [95% CI: 30.4, 39.2]), European umbrella sports federations (38.5% [95% CI: 12.0, 64.9]), the organisations that were highly committed to the promotion of elite sports (34.0% [95% CI: 29.4, 38.6]) and the organisations whose representatives were not aware of the SCforH guidelines (35.7% [95% CI: 31.1, 40.3]).

**Table 1:** The commitment of sports organisations in Europe to the promotion of health-enhancing physical activity (HEPA)

Category	*n*^a^ (%)	Commitment to HEPA promotion; % (95% CI)^b^			
		**Low**	**Medium**	**High**	***p*** ^c^
***Overall sample***	536 (100)	32.1 (28.1, 36.0)	39.7 (35.6, 43.9)	28.2 (24.4, 32.0)	< 0.001
***Type of organisation***					
National sport associations	451 (84.1)	34.8 (30.4, 39.2)	42.1 (37.6, 46.7)	23.1 (19.2, 26.9)	< 0.001
European umbrella sports federations	13 (2.4)	38.5 (12.0, 64.9)	30.8 (5.7, 55.9)	30.8 (5.7, 55.9)	
National umbrella sports organisations	12 (2.2)	25.0 (0.5, 49.5)	25.0 (0.5, 49.5)	50.0 (21.7, 78.3)	
National Olympic committees	20 (3.7)	20.0 (2.5, 37.5)	25.0 (6.0, 44.0)	55.0 (33.2, 76.8)	
National sport-for-all organisations	40 (7.5)	7.5 (-0.7, 15.7)	27.5 (13.7, 41.3)	65.0 (50.2, 79.8)	
***European Union***					
No	68 (12.7)	32.4 (21.2, 43.5)	45.6 (33.8, 57.4)	22.1 (12.2, 31.9)	0.430
Yes	468 (87.3)	32.1 (27.8, 36.3)	38.9 (34.5, 43.3)	29.1 (24.9, 33.2)	
***Region*** ^d^					
Western Europe	148 (27.6)	37.2 (29.4, 44.9)	35.8 (28.1, 43.5)	27.0 (19.9, 34.2)	0.089
Central and Eastern Europe	145 (27.1)	26.2 (19.0, 33.4)	42.1 (34.0, 50.1)	31.7 (24.1, 39.3)	
Northern Europe	155 (28.9)	34.2 (26.7, 41.7)	44.5 (36.7, 52.3)	21.3 (14.8, 27.7)	
Southern Europe	88 (16.4)	29.5 (20.0, 39.1)	34.1 (24.2, 44.0)	36.4 (26.3, 46.4)	
***Commitment to elite sports***					
Low	55 (10.3)	25.5 (13.9, 37.0)	29.1 (17.1, 41.1)	45.5 (32.3, 58.6)	0.031
Medium	78 (14.6)	26.9 (17.1, 36.8)	41.0 (30.1, 51.9)	32.1 (21.7, 42.4)	
High	403 (75.2)	34.0 (29.4, 38.6)	40.9 (36.1, 45.7)	25.1 (20.8, 29.3)	
***Awareness of SCforH*** ^e^ ***guidelines***					
No	420 (78.4)	35.7 (31.1, 40.3)	41.0 (36.2, 45.7)	23.3 (19.3, 27.4)	< 0.001
Yes	116 (21.6)	19.0 (11.8, 26.1)	35.3 (26.6, 44.0)	45.7 (36.6, 54.8)	

a Number of sports organisations

b Percentage of sports organisations with a low, medium, or high level of commitment to the promotion of HEPA and its 95% confidence interval

c P-value from the Fisher’s exact test

d Region of Europe according to EuroVoc

e Sports Club for Health

The multiple linear regression analysis, adjusted for all independent variables in the model, showed that the commitment of sports organisations to HEPA promotion is associated with the type of organisation, the region of Europe in which the organisation was located, and the awareness of SCforH guidelines (Table 2). The national Olympic committees (*β* = 1.48 [95% CI: 0.41, 2.55], *p* = 0.007) and the national sport-for-all organisations (*β* = 1.68 [95% CI: 0.74, 2.62], *p* < 0.001) were significantly more committed to HEPA promotion than national sport associations (ref). The sports organisations in Central and Eastern Europe were significantly more committed to HEPA promotion, compared with the sports organisations in Western Europe (*β* = 0.56 [95% CI: 0.01, 1.12], *p* = 0.047). The awareness of SCforH guidelines was associated with a higher commitment of the sports organisation to HEPA promotion (*β* = 0.86 [95% CI: 0.35, 1.37], *p* < 0.001).

**Table 2:** Correlates of the commitment of sports organisations in Europe to the promotion of health-enhancing physical activity (HEPA): results of a multiple linear regression analysis

Independent variables	*β* (95% CI)^a^	*p* ^b^
***Type of organisation***		
National sport associations	*Ref* ^c^	
European umbrella sports federations	0.86 (-0.48, 2.20)	0.206
National umbrella sports organisations	0.51 (-0.87, 1.89)	0.471
National Olympic committees	1.48 (0.41, 2.55)	0.007
National sport-for-all organisations	1.68 (0.74, 2.62)	< 0.001
***European Union***		
No	*Ref* ^c^	
Yes	-0.17 (-0.79, 0.44)	0.577
***Region*** ^d^		
Western Europe	*Ref* ^c^	
Central and Eastern Europe	0.56 (0.01, 1.12)	0.047
Northern Europe	0.11 (-0.43, 0.65)	0.696
Southern Europe	0.40 (-0.23, 1.03)	0.216
***Commitment to elite sports***		
Low	*Ref* ^c^	
Medium	0.10 (-0.80, 1.00)	0.834
High	-0.42 (-1.23, 0.38)	0.305
***Awareness of SCforH*** ^e^ ***guidelines***		
No	*Ref* ^c^	
Yes	0.86 (0.35, 1.37)	<0.001

a Unstandardized regression coefficient adjusted for all independent variables listed in the table and its 95% confidence interval

b P-value for the unstandardized regression coefficient

c Reference group

d Region of Europe according to EuroVoc

e Sports Club for Health

Compared with national sports organisations, European umbrella sports federations had a higher commitment to the promotion of health-enhancing sports, while national Olympic committees had a higher commitment to the promotion of health-enhancing exercise and health-enhancing lifestyle physical activities (Table 3). National sport-for-all organisations and organisations whose representatives were aware of the SCforH guidelines had a higher commitment to all three types of HEPA. Compared with sports organisations from Western Europe, the organisations from Central and Eastern Europe and Southern Europe had a higher commitment to the promotion of health-enhancing lifestyle physical activities.

**Table 3:** Correlates of the commitment of sports organisations in Europe to the promotion of health-enhancing sports activity (HESA), health-enhancing exercise (HEXE), and health-enhancing lifestyle physical activities (HELPA): results of three multiple ordinal logistic regression analyses

Independent variables	HESA		HEXE		HELPA	
	**OR (95% CI)** ^a^	***p*** ^b^	**OR (95% CI)** ^a^	***p*** ^b^	**OR (95% CI)** ^a^	***p*** ^b^
**Type of organisation**						
National sport associations	*Ref* ^c^		*Ref* ^c^		*Ref* ^c^	
European umbrella sports federations	3.70 (1.26, 11.71)	0.019	0.85 (0.29, 2.48)	0.771	1.61 (0.54, 4.69)	0.380
National umbrella sports organisations	1.72 (0.58, 5.28)	0.332	2.14 (0.73, 6.08)	0.156	0.95 (0.35, 2.53)	0.913
National Olympic committees	2.06 (0.89, 4.86)	0.092	3.02 (1.31, 7.09)	0.010	2.82 (1.27, 6.32)	0.011
National sport-for-all organisations	3.17 (1.52, 6.78)	0.002	3.56 (1.74, 7.43)	0.001	2.44 (1.19, 5.04)	0.015
**European Union**						
No	*Ref* ^c^		*Ref* ^c^		*Ref* ^c^	
Yes	1.03 (0.66, 1.61)	0.884	0.84 (0.54, 1.30)	0.435	0.81 (0.52, 1.29)	0.376
**Region** ^d^						
Western Europe	*Ref* ^c^		*Ref* ^c^		*Ref* ^c^	
Central and Eastern Europe	1.21 (0.80, 1.82)	0.371	1.36 (0.90, 2.05)	0.142	1.75 (1.16, 2.64)	0.008
Northern Europe	1.40 (0.93, 2.11)	0.103	0.98 (0.65, 1.46)	0.908	0.95 (0.63, 1.42)	0.787
Southern Europe	1.06 (0.66, 1.69)	0.817	1.13 (0.71, 1.81)	0.610	1.67 (1.03, 2.69)	0.037
**Commitment to elite sports**						
Low	*Ref* ^c^		*Ref* ^c^		*Ref* ^c^	
Medium	0.79 (0.40, 1.57)	0.503	0.87 (0.44, 1.70)	0.675	1.15 (0.60, 2.20)	0.681
High	0.94 (0.49, 1.75)	0.837	0.65 (0.35, 1.20)	0.173	0.64 (0.35, 1.15)	0.133
**Awareness of SCforH** ^e^ **guidelines**						
No	*Ref* ^c^		*Ref* ^c^		*Ref* ^c^	
Yes	1.48 (1.01, 2.19)	0.047	1.82 (1.24, 2.67)	0.002	1.78 (1.21, 2.61)	0.003

a Odds ratio adjusted for all independent variables listed in the table and its 95% confidence interval

b P-value for the odds ratio

c Reference group

d Region of Europe according to EuroVoc

e Sports Club for Health

## Discussion

### Key findings

The main finding of our study is that less than one third of sports organisations in Europe are highly committed to HEPA promotion. We also found that a higher commitment to HEPA promotion is associated with the national Olympic committees, national sport-for-all organisations, sports organisations from the Central and Eastern Europe, and the awareness of SCforH guidelines. Most findings for the commitment of sports organisations to specific types of HEPA were in accordance with the findings for overall HEPA.

### Level of commitment to HEPA promotion

Our findings suggest that the potential for health promotion through sports organisations is still underutilised. It may be that sports clubs lack the necessary resources, such as funding, adequate facilities, volunteers, and staff, to effectively implement both HEPA and elite sport programmes [20]. Consequently, they may be unable to provide the necessary opportunities for widespread community involvement in their activities [20]. It has been suggested that prioritising investments in elite sports may have a negative impact on investments in ‘sport for all’ [29]. Also, the historical orientation of sports organisations to professional sports and achieving their core “obligation” of winning medals in competitions [29, 30] may limit their commitment to ‘sport for all’.

With sports for health becoming more and more important topic on the political agenda, the complementarity between elite sport development and the promotion of ‘sport for all’ is increasingly discussed [28]. The complementarity of elite sports and ‘sport for all’ assumed in the “virtuous cycle of sport” and the “pyramid theory” has been questioned [28, 44]. While some authors have put forward arguments for a divergent development of elite sports and ‘sport for all’ [44], others suggest there is evidence of some complementarity between the two [28]. Nevertheless, striking the right balance between the investments in elite sport and ‘sport for all’ is needed to improve HEPA promotion, regardless of the level of their complementarity.

Previous research has shown that SCforH programmes were implemented in only seven EU countries in 2015 [45] and in only six EU countries in 2018 [46], which may partially explain the relatively low percentage of European sports organisations in our sample that were highly committed to HEPA promotion. While European Union policies emphasise the importance of HEPA promotion through sports clubs and organisations, it may be that this has not been adequately addressed in national-level policies in all member states. Improvements in national physical activity policies may be needed to facilitate the promotion of HEPA through sports organisations. It is worth emphasising that several factors may influence the development, implementation, and impact of sport policies in a given country, and that they may differ between countries, making policy convergence a challenging task [47]. Differences in national policies and structure of the sports system may explain variability in sport participation rates across different countries [48]. Therefore, when developing national policies relevant to HEPA promotion through sports clubs, policymakers should consider examples of good policies and organisational structures from the countries with higher sport participation rates.

### Correlates of the commitment of sports organisations to HEPA promotion

We found that the organisations from Central and Eastern Europe have a higher overall commitment to HEPA promotion than the sports organisations from Western Europe, while the organisations from Southern Europe had a high commitment to health-enhancing sports activity. This is in contrast to the findings of Breuer et al. [17] study suggesting that the Central and Eastern European as well as Southern countries are oriented more towards elite sports and less towards other benefits and values of sports, compared with the Western European countries. However, it should be noted that the Breuer et al. [17] study included only four Central and Eastern European countries; namely, Czech Republic, Hungary, Poland, and Slovenia, and only three Southern countries: Greece, Italy, and Spain. It may be that our findings are different because they reflect the situation in a wider range of countries in the region. During the communist era in these countries, sport was controlled exclusively by the governments, and, according to Breuer et al. [17], they favoured elite sport and used it to build their country’s international reputation. However, after the World War II, the “Soviet concept of physical culture” was also very popular in this European region [49]. The concept addressed population health and recreation through physical education, health literacy, hygiene, competitive sport, and sport for all [50]. It is possible that sports organisations in Central and Eastern Europe inherited these historical values, which would explain their higher commitment to HEPA promotion found in our study. From our analyses, it seems that the higher overall commitment of sports organisations from Central and Eastern Europe to HEPA is mainly due to their higher commitment to health-enhancing lifestyle physical activities.

Our findings also suggest that the national Olympic committees and sport-for-all organisations have the highest overall commitment to HEPA promotion, while the European umbrella sports federations had a high commitment to the promotion of health-enhancing sports activity. This was expected due to their jurisdiction and scope of activities. For example, the primary vision of The Association For International Sport for All (TAFISA), which is reflected in the visions of many national sport-for-all organisations, is that all people should have access to physical activity that is necessary to achieve a healthy lifestyle [51]. The national Olympic committees operate in accordance with the recent Olympic agenda that recommends to strengthen the role of sports in reaching the UN Sustainable Development Goals by supporting social and health development through increased sports participation [33]. Another possible explanation for the higher commitment of national Olympic committees to HEPA promotion is that for larger organisations it may be easier commit to both elite and recreational sports, due to their available resources (e.g., membership, funding, and employed staff) [52]. A similar assumption was also made when comparing HEPA promotion in larger and smaller sports clubs [17]. There is a widely held belief that hosting major sporting events and having national teams that perform well at such events would facilitate higher sport participation in the population [28]. However, the empirical evidence to support this belief is questionable [28]. In their attempt to increase sports participation in the population, it is possible that Olympic committees therefore put increased emphasis on alternative strategies, such as promoting HEPA through sports clubs.

The association between the awareness of SCforH guidelines and a higher commitment of sports organisations to HEPA promotion indicates the importance of disseminating the SCforH guidelines in Europe and confirms the significance of this indicator in the Council Recommendations. This is in accordance with previous findings from the public health sector showing that practical guidelines and initiatives can lead to positive changes [53, 54]. Policymakers should aim to improve the commitment of sports organisations to HEPA promotion by issuing policies and increasing funding that would support a wide adoption of the SCforH approach.

### Implications for policy and practice

Our findings may inform the development and/or refinement of EU- and national-level physical activity policies and practices of sports organisations in relation to HEPA promotion. In specific, national Olympic committees and sport-for-all organisations can be used as models for HEPA promotion in other types of sports organisations. This should be done by taking into consideration that their approaches to HEPA promotion may need to be adapted to better align with the aims and scope of other types of sports organisations. A number of examples of good practice of HEPA promotion through sports organisations are likely to be found among the countries in Central and Eastern Europe. However, it should be taken into account that the way HEPA promotion through sports organisations is facilitated should be tailored to the specific political, socioeconomic, and cultural context in the given country. The commitment of sports organisations to HEPA promotion could also be increased by raising the awareness and utilisation of SCforH guidelines among their representatives. The recommended approaches for implementation of SCforH guidelines in sports organisations have been described elsewhere [5, 55].

### Strengths and limitations

The key strengths of this study include: (1) quantitative assessment of the commitment of sports organisations to promoting different types of physical activity, which allowed us to analyse its correlates; (2) study sample that included the representatives of sports organisations, which ensured that the participants have adequate knowledge and/or access to information needed to complete the survey; and (3) large and diverse sample size including 536 sports organisations from 36 European countries, which allowed us to make comparisons by the type of organisation and by the region and EU membership of the country in which the organisation is located.

The study had four key limitations. First, its cross-sectional design prevented drawing conclusions about the direction of causality between the variables. For example, it is possible that a higher awareness of SCforH guidelines was either a cause or a consequence of a higher commitment to the HEPA promotion, or that the relationship between these variables was bidirectional. Our findings should therefore be taken with caution and further investigated in longitudinal and intervention studies. Second, other characteristics of sports organisations that were not assessed in our survey may be associated with the commitment to HEPA promotion. Therefore, there is a possibility that our findings are affected by residual confounding. Future studies on this topic should aim to include a wider range of explanatory variables in their analyses. Third, the study sample did not include sports organisations from all European countries, which may limit the generalisability of our findings. Fourth, the level of commitment to specific types of physical activity may vary across different countries. However, we could not include all countries as independent variables in the regression model, because our sample was too small and that would significantly increase the probability of type 2 error. Therefore, we grouped countries into four regions.

## Conclusion

From our findings, it seems that most sports organisations are highly committed to elite sports. Only one third of sports organisations in Europe are highly committed to HEPA promotion. Given that increasing the population levels of physical activity is one of the key public health priorities in Europe, coordinated actions at the EU and national levels are needed to improve the promotion of HEPA through sports organisations. This should include various stakeholders in the sports sectors, such as representatives of sports clubs and associations, HEPA researchers and promoters, policymakers in the areas of health and sport, and tertiary education teachers and students of sport and exercise science, physical education, and health promotion. In this endeavour, it may be useful to consider national Olympic committees, national sport-for-all organisations, and relevant sports organisations in Central and Eastern Europe as role models and raise the awareness of SCforH guidelines among the representatives of sports organisations. Future research should examine other possible strategies to facilitate HEPA promotion through sports organisations, especially initiatives by policymakers at the EU and national levels aimed to improve sport policies and ways to ensure a better balance between funding for elite sports and ‘sport for all’.

## Data Availability

According to the conditions of the ethics approval, the data used in this study cannot be shared publicly. The data will be shared upon a reasonable request sent to the corresponding author.

## Abbreviations

EU: European Union
HEPA: Health-enhancing physical activity
HESA: Health-enhancing sports activity
HEXE: Health-enhancing exercise
HELPA: Health-enhancing lifestyle physical activities
SCforH: Sports Club for Health
